# Does past evolutionary history under different mating regimes influence the demographic dynamics of interspecific competition?

**DOI:** 10.1002/ece3.5397

**Published:** 2019-07-05

**Authors:** Daisuke Kyogoku, Michio Kondoh, Teiji Sota

**Affiliations:** ^1^ Ecological Integration, Graduate School of Life Sciences Tohoku University Aoba Sendai Japan; ^2^ Department of Science and Technology Ryukoku University Seta Otsu Japan; ^3^ Department of Zoology, Graduate School of Science Kyoto University Sakyo Kyoto Japan; ^4^ Research Institute for Food and Agriculture Ryukoku University Otsu Shiga 520‐2194 Japan

**Keywords:** *Callosobruchus*, eco‐evolutionary dynamics, empirical dynamic modeling, experimental evolution, interspecific competition, reproductive interference

## Abstract

Interspecific interactions are contingent upon organism phenotypes, and thus phenotypic evolution can modify interspecific interactions and affect ecological dynamics. Recent studies have suggested that male–male competition within a species selects for capability to reproductively interfere with a closely related species. Here, we examine the effect of past evolutionary history under different mating regimes on the demographic dynamics of interspecific competition in *Callosobruchus* seed beetles. We used previously established experimental evolution lines of *Callosobruchus chinensis* that evolved under either forced lifelong monogamy or polygamy for 17 generations, and examined the demographic dynamics of competition between these *C. chinensis* lines and a congener, *Callosobruchus maculatus*. *Callosobruchus chinensis* was competitively excluded by *C. maculatus* in all trials. Time series data analyses suggested that reproductive interference from *C. chinensis* was relatively more important in the trials involving polygamous *C. chinensis* than those involving monogamous *C. chinensis*, in accordance with the potentially higher reproductive interference capability of polygamous *C. chinensis*. However, the estimated signs and magnitudes of interspecific interactions were not fully consistent with this explanation, implying the evolution of not only reproductive interference but also other interaction mechanisms. Our study thus suggests multifaceted effects of sexually selected traits on interspecific competitive dynamics.

## 1 INTRODUCTION

A tenet of community ecology is that the phenotypes of organisms determine the characteristics (e.g., mode, sign, or intensity) of interspecific interactions. Different beak sizes of Darwin's finches allow them to forage different food items, achieving niche segregation (Grant & Grant, 2006; De León, Podos, Gardezi, Herrel, & Hendry, 2014). Snail‐eating snakes show laterally asymmetric adaptations in morphology and behavior that allow them to eat snails with the more common chirality, which in turn enables snails with less common chirality to escape predation by these snakes (Hoso et al., 2010). Phenotype‐dependence of interspecific interaction means that phenotypic evolution can modify the intensity of interspecific interactions and thus affect ecological dynamics (Yoshida, Jones, Ellner, Fussmann, & Hairston NGJr, 2003).

A potential interaction that may occur between a pair of closely related species is reproductive interference. Males are indiscriminate in many taxa, and consequently heterospecific mating interactions, such as persistent interspecific courtship, interspecific copulation with poor mechanical matching, or hybridization can reduce the population growth of the species involved (Burdfield‐Steel & Shuker, 2011; Gröning & Hochkirch, 2008; Kyogoku, 2015). Recent studies have reported cases wherein such reproductive interference plays an important role in species coexistence (Crowder et al., 2010; Kishi, Nishida, & Tsubaki, 2009; Liu et al., 2007; Takakura, Nishida, Matsumoto, & Nishida, 2009).

Some empirical studies have suggested that sexual selection underlies the evolution of reproductive interference (Kyogoku & Sota, 2015, 2017; Yassin & David, 2016). Male–male competition over mating or fertilization within a species can select for male adaptations that increase his mating or fertilization success at the cost of the fitness of his mates (Arnqvist & Rowe, 2005; Parker, 1979). Examples of such male adaptations include spiny penis (Crudgington & Siva‐Jothy, 2000; Rönn, Katvala, & Arnqvist, 2007), toxic ejaculate (Chapman, Liddle, Kalb, Wolfner, & Partridge, 1995; Wigby & Chapman, 2005), and aggressive male behavior (Le Galliard, Fitze, Ferrière, & Clobert, 2005; Réale, Boussès, & Chapuis, 1996; Sugano & Akimoto, 2011). If mating interactions occur between species, the harmful male traits can damage heterospecific females. In other words, reproductive interference can at least in part be a side effect of sexually selected harmful male traits.

The strength of male–male competition is dependent on the potential of females to mate with multiple males; if females encounter only a single male during their lives, male–male competition does not occur, whereas the presence of many rival males will select for male adaptations to outcompete rival males. Some experimental studies have enforced lifelong monogamy on naturally polygamous organisms to manipulate the strength of sexual selection (e.g., Holland & Rice, 1999; Pitnick, Miller, Reagan, & Holland, 2001; Martin & Hosken, 2003; Cayetano, Maklakov, Brooks, & Bonduriansky, 2011; Gay, Hosken, Eady, Vasudev, & Tregenza, 2011). Enforced lifelong monogamy eliminates the opportunity for male–male competition, and thus for sexual selection. Such studies have identified evolutionary responses in reproductive traits in accordance with theoretical predictions.

We predict that evolution under environments with different propensities for female multiple mating may, via its effect on sexual selection and reproductive interference, affect the dynamics of subsequent interspecific competition. However, evolution of certain types of behavioral interactions (e.g., reproductive interference) that act at individual level may have little effects on demographic dynamics. Here, our interest is not in the evolution of certain types of behavioral interactions but in their net causal effects on demographic dynamics at population level. For example, evolution of reproductive interference capability may change the time until competitive exclusion. Alternatively, strong reproductive interference may destabilize the trajectory of demographic dynamics, for example, potentially leading to multimodal distribution of the time until competitive exclusion. Furthermore, these predictions assume the evolution of between‐population demographic interactions or causal effects at population level. Recent advances in empirical dynamic modeling (EDM) enable to examine the demographic interactions between populations. EDM is a model‐free framework for time series data analysis (Deyle et al., 2013, Deyle, May, Munch, & Sugihara, 2016, Ye, Deyle, Gilarranz, & Sugihara, 2015). Within this framework, convergent cross‐mapping (CCM) technique can identify causal effects from one population to another (Sugihara et al., 2012). Subsequent application of multivariate S‐map (sequential locally weighted global linear map; Deyle et al., 2016) quantifies the demographic interactions. Therefore, EDM enables the comparison of dynamic characteristics of competition involving populations with different evolutionary histories.

*Callosobruchus chinensis* and *Callosobruchus maculatus* have served as a laboratory model system for reproductive interference (Kishi, 2015). Males of both species indiscriminately try to mate with conspecific and heterospecific females (Kishi et al., 2009; Shimomura, Mimura, Ishikawa, Yajima, & Ohsawa, 2010). Interspecific copulation between *C. maculatus* females and *C. chinensis* males occurs repeatedly and reduces female fecundity by physically damaging female genitalia (Kyogoku & Sota, 2015). This reproductive interference by *C. chinensis* males with *C. maculatus* females appears to be intensified by sexual selection on the former (Kyogoku & Sota, 2015, 2017). Males of *Callosobruchus* seed beetles have sexually selected genital spines (e.g., Hotzy & Arnqvist, 2009; Hotzy, Polak, Rönn, & Arnqvist, 2012). A study that exploited between‐population phenotypic variance suggested that the genital spines induce the genital damage via interspecific copulation, causing the reproductive interference (Kyogoku & Sota, 2015). Kishi et al. (2009) showed that, at least in a certain combination of beetle strains, competitive exclusion of *C. maculatus* by *C. chinensis* within a few generations could be attributed to this reproductive interference, but not to larval resource competition, in which *C. maculatus* was dominant to *C. chinensis*. There are many other studies reporting the extinction of *C. maculatus* in competition experiments with *C. chinensis* (Kishi, 2015).

A previous study examined the evolution of reproductive interference capability by sexual selection using this system (Kyogoku & Sota, 2017). They imposed enforced lifelong monogamy or ancestral polygamy to replicated experimental evolution lines of *C. chinensis* for 17 generations. At postevolution reproductive interference assay at individual level, polygamous *C. chinensis* interfered with the reproduction of *C. maculatus* more strongly than monogamous *C. chinensis*. However, male genital morphology did not show any significant divergence between polygamous and monogamous lines. Therefore, traits other than genital morphology likely have underlain the evolution of reproductive interference. For example, sexual selection under polygamy may have selected for active locomotion (e.g., to encounter females frequently; see also Martinossi‐Allibert, Thilliez, Arnqvist, & Berger, 2018). Because frequent heterospecific encounter and consequently frequent interspecific copulation intensifies reproductive interference (Kyogoku & Nishida, 2013), it is possible that the locomotor activity underlies the evolution of reproductive interference capability under polygamy. This experimental evolution lines offer an opportunity to examine how evolutionary history affects the demographic dynamics of interspecific competition.

Here, we examine the effects of past evolutionary history under different mating regimes on interspecific competitive dynamics, using the previously established experimental evolution lines (polygamy and monogamy lines) of *C. chinensis* and a reference line of *C. maculatus*. We test two hypotheses using this experimental system. First, we test if sexual selection on *C. chinensis* leads to different outcomes of interspecific competition. Under laboratory conditions, the predominant mode of interaction between these two species is reproductive interference during the adult stage and resource competition during the larval stage (Kawatsu & Kishi, 2018). Although sexual selection may intensify reproductive interference, there is no clear prediction that resource competition should be affected by short‐term evolutionary responses to sexual selection. Therefore, sexual selection may change the relative contributions of reproductive interference and resource competition to competitive dynamics. For example, whereas sexually selected *C. chinensis* may outcompete *C. maculatus*, sexually nonselected *C. chinensis* may be outcompeted by *C. maculatus*. Alternatively, sexually selected *C. chinensis* may drive *C. maculatus* extinct more rapidly than sexually nonselected *C. chinensis*. Similarly, if *C. chinensis* is outcompeted by *C. maculatus*, sexually selected *C. chinensis* may persist longer than sexually nonselected *C. chinensis*. We analyzed the time until extinction to determine whether evolutionary history of a *C. chinensis* population affected the persistence of populations during interspecific competition. Second, we examined whether characteristics of interspecific interactions had evolved due to sexual selection, by testing the prediction that sexually selected and nonselected *C. chinensis* may show different intensities of interspecific interactions during competition with *C. maculatus*. Specifically, we performed CCM to determine the causal effect from one species to the other and their time lags. In the *Callosobruchus* competition system, larval resource competition and adult reproductive interference show different time lags (Kawatsu & Kishi, 2018), and thus time lag can be used to infer the behavioral mechanisms of the interactions. We then performed S‐map to quantify the intensity of the interspecific interactions.

## 2 MATERIAL AND METHODS

### 2.1 Study organisms

Both *C. chinensis* and *C. maculatus* are pests of postharvest *Vigna* beans. Larvae grow inside a dry bean, which the larvae eat. Generation time is approximately 3 weeks at 30°C. Adults are sexually mature at emergence, and they can mate and lay eggs without food or water. Hybrids are not produced between them. During reproduction, males are not engaged in direct male–male competition or mate guarding. Both males and females can mate multiply, and the natural mating system is promiscuous (Miyatake & Matsumura, 2004; Harano & Miyatake,^2005^). We used adzuki beans, *Vigna angularis*, for larval food. Experiments were performed under laboratory conditions of 30°C, relative humidity 70%, and 16L8D, unless otherwise noted.

### 2.2 Experimental evolution lines

We performed experimental evolution using *C. chinensis* for 17 generations, details of which have been described elsewhere (Kyogoku & Sota, 2017). Briefly, we mixed nine different populations of *C. chinensis* to ensure additive generic variation, from which we derived six replicated evolutionary lines (40 males and 40 females for each). Three lines were maintained under forced lifelong monogamy, whereas the other three lines were maintained under polygamy. Factors other than mating regime, such as resource availability, were comparable among the lines. After 17 generations of experimental evolution, we maintained the lines under common garden conditions of lifelong monogamy for two generations before the following competition experiment.

### 2.3 Interspecific competition experiments

We performed replicated competition experiments using a reference *C. maculatus* line (hQ; Miyatake & Matsumura, 2004) and the evolutionary lines of *C. chinensis*. The experimental method followed Kishi et al. (2009): generations were made to overlap by introducing adult beetles into the arena multiple times; new beans were provided every week and the beans were discarded after 4 weeks. We used Petri dishes with four compartments, where adult beetles were able to walk across the compartments. We used four pairs per species (i.e., the equal initial abundance) for starting each experiment based on the results of Kishi et al. (2009), in which *C. chinensis* always excluded *C. maculatus*. On day 1, we introduced four males and four females of each species into an empty dish. We also introduced 5 g of adzuki beans into one compartment. On days 8 and 15, we similarly introduced beetles and beans into the dish, using new compartments for the beans each day. On day 22, we introduced only beans. Starting on day 29, we replaced the oldest beans with new beans every 7 days. At the time of bean replacement, we lightly anesthetized beetles with diethyl ether, removed dead beetles, and recorded the presence/absence of adults of each species. We continued to replace the beans until either species became extinct, which we defined as the absence of adults for 5 consecutive weeks. We made six replicates for each of the six *C. chinensis* evolutionary lines. For time series analysis, we counted the number of beetles at bean replacements for four replicates: two involving a polygamous *C. chinensis* line and the other two involving a monogamous line. We used virgin beetles within 72 hr after emergence. Beetles were individually stored and kept at 20°C until use to reduce exhaustion.

### 2.4 Data analyses

We first examined the effects of the selection regime on the outcome of interspecific competition. The effect on the mean time until extinction was analyzed using a linear mixed model (LMM), where mating regime and line replicates were included as fixed and random effects, respectively. We used the reciprocal of time until competitive exclusion as the response variable to normalize the distribution. We also performed survival analysis, which is a method to analyze the factors affecting the time until some event happens, such as recovery from a disease or death in clinical trials. We applied this method to analyze the “mortality” of competition replicates or the probability of extinction in either species. Survival analysis compares not means but distributions and may find a difference that does not change the mean. In particular, we used the Cox model, which assumes that hazard functions (probability of extinction of survivors in a unit time) are proportional between treatments. We examined whether the hazard functions of polygamous and monogamous treatments were proportional over time. Mating regime and line replicates were included as fixed and random effects, respectively.

To examine the strength of interspecific interactions during the competition experiment, we analyzed the time series of census data using EDM, which is based on the state space reconstruction, for example, from a single time series with lagged coordinate embedding: **x*_t_*** = {*x*(*t*), *x*(*t* − *τ*), *x*(*t* − 2*τ*),…, *x*(*t* − (*E* − 1)*τ*)}, where *x*(*t*) is the value of variable *x* at time *t*, *τ* is the embedding lag and *E* is the embedding dimension. EDM is an analysis method for deterministic, nonlinear systems (Chang, Ushio, & Hsieh, 2017). For the applicability of EDM to *C. chinensis*‐*C. maculatus* competitive dynamics, see Kawatsu and Kishi (2018). Following the ordinary procedure, we first performed simplex projection to determine the embedding dimension *E*, the embedding lag *τ* and the number of time steps to predict *T*_P_. We then examined the demographic interactions between *C. chinensis* and *C. maculatus* by performing cross‐mapping. We evaluated the convergence of cross‐mapping skill by nonparametric bootstrap and searched the optimal time lag for prediction *l*. We also estimated the rate of false positive by cross‐mapping our data to that of Kishi et al. (2009). Finally, we quantified the intensities of interspecific interactions via the application of S‐map to multivariate reconstructed state spaces. S‐map analysis predicts the future state of the system by regression in state space, where data points are weighted depending on their distance from the predictee. S‐map gives the regression coefficient, which is a measure of how sensitively the predictee changes with slight changes in a focal variable for each time point (i.e., an element of Jacobian). The effect of one variable on another variable changes depending on the state of the system in nonlinear systems. We pooled estimated S‐map coefficients and compared their distributions between treatments. We used *F* and *t* tests to compare the variance and mean, respectively, using the Holm‐Bonferroni method for multiple comparisons. For pairs that showed a significant difference in variance, we used Welch's method to adjust the degree of freedom of the *t* test. The details of EDM analysis are available in the Supplementary material. All data analyses were performed using R software (ver. 3.5.1; 2018, Vienna, Austria). For EDM analysis, we used the rEDM package (ver. 0.7.2).

## 3 RESULTS

**Figure 1:**
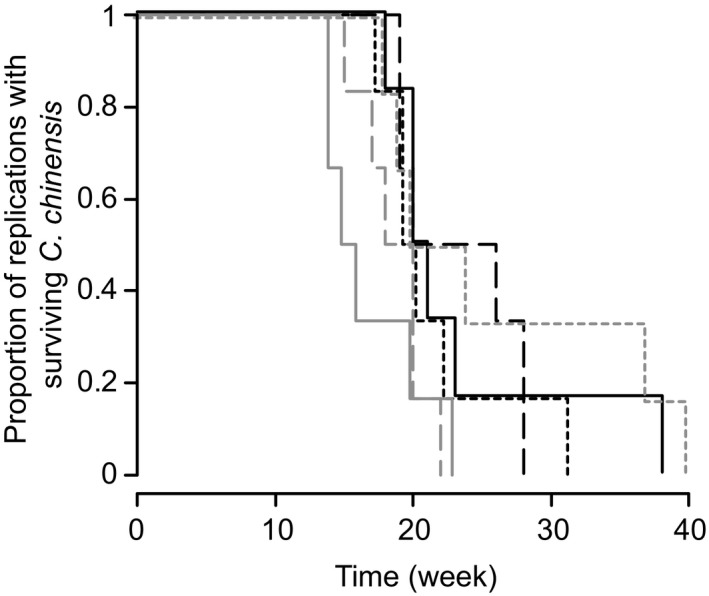
Persistence of *Callosobruchus chinensis* evolutionary lines competing with *Callosobruchus maculatus*. Survivorship, which is defined as the proportion of surviving lines out of six replicates, is shown against time. Gray lines represent polygamous *C. chinensis* lines, and black lines represent monogamous lines. Solid, dashed, and dotted lines represent independent evolutionary lines (i.e., replicates)

**Figure 2:**
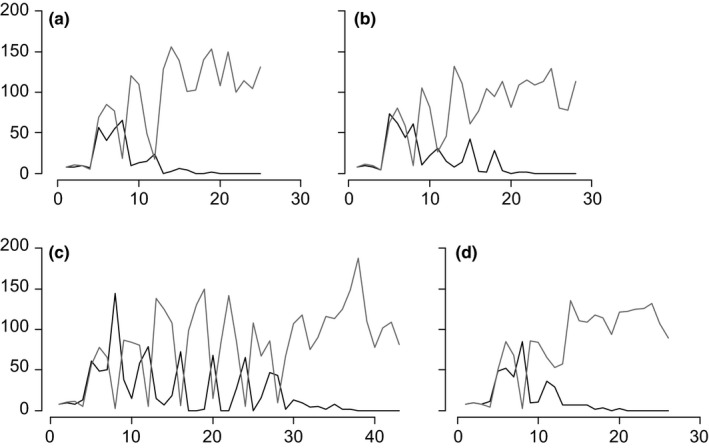
Demographic dynamics of *Callosobruchus chinensis* (black lines) and *Callosobruchus maculatus* (gray lines) during the interspecific competition that was used for empirical dynamic modeling (EDM) analysis. (a) and (b) competition involving a polygamous *C. chinensis* line. (c) and (d) competition involving a monogamous *C. chinensis* line

All replicates of the competition experiment resulted in the extinction of *C. chinensis*, which occurred within 14–40 weeks (Figures 1 and 2). *Callosobruchus chinensis* persisted for 17–37 weeks (mean: 22.7 weeks) for monogamous lines and 14–40 weeks (mean: 20.7 weeks) for polygamous lines. Mean persistence time did not differ significantly between polygamous and monogamous lines (Wald *t* test of LMM: *t* = 1.11, *p* = 0.33). However, survival analysis suggested that the distributions of extinction timing may have been different between treatments; the extinction risk ratio (polygamous lines: monogamous lines) was marginally significantly dependent on time (Schoenfeld residuals test: *ρ* = −0.285, *χ* ^2^ = 3.84, *p* = 0.05; Figure S1). This indicates that the extinction risk ratio of polygamous lines compared to monogamous lines may have been higher at early stages of competition than later.

**Figure 3:**
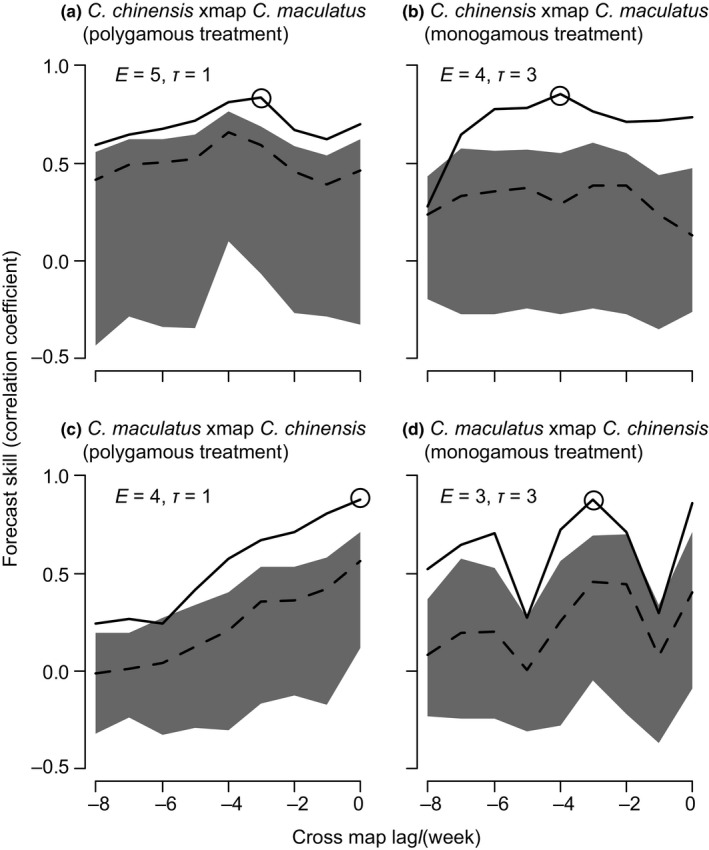
Convergent cross‐mapping for interspecific interactions. Solid lines give the forecast skill with the maximum library size (all data) under a given cross map lag *l*. Optimal cross map lag, which shows the highest forecast skill, is indicated by a circle in each panel. Dashed lines give the median forecast skill with the minimum library sizes (*E* + 1). Gray areas give 95% confidence interval of the forecast skill with the minimum library sizes

**Figure 4:**
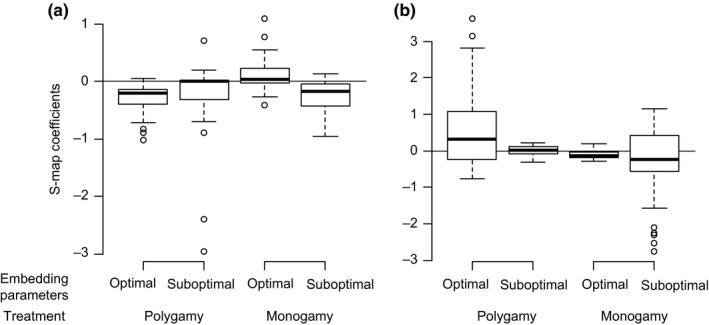
Sequential locally weighted global linear map (S‐map) coefficients for interspecific interactions. Two S‐map analyses with different parameters (*E*, *T_P_*, *l*) were performed for each species‐treatment combination. “Optimal” parameters are optimized for the focal treatment of the focal species. “Suboptimal” parameters are those optimized for the focal species, but of the other treatment. The top and bottom of a box represent 25% and 75% quartiles, and the bold line in a box represents the median. Whiskers extend to the most extreme data points within a 1.5 quartile range, and dots are data points beyond the whiskers. (a) effects of *Callosobruchus maculatus* on *Callosobruchus chinensis* (optimal for polygamy: *E* = 5, *τ* = 1, *T_P_* = 3, *l* = −3; optimal for monogamy: *E* = 4, *τ* = 3, *T_P_* = 4, *l* = −4). (b) effects of *C. chinensis* on *C. maculatus* (optimal for polygamy: *E* = 4, *τ* = 1, *T_P_* = 4, *l* = 0; optimal for monogamy: *E* = 3, *τ* = 3, *T_P_* = 1, *l* = −3)

To examine the characteristics of interspecific interactions, we applied EDM to analyze the time series of population dynamics. Optimal embedding parameters (e.g., number of time steps necessary to predict future time steps) were dependent on both species and mating regime treatment (Figure S2). In the univariate simplex projection for polygamous *C. chinensis*, the forecast skill was highest with the embedding dimension *E* = 5, the embedding lag *τ* = 1, and the time to predict *T_P_* = 3. For monogamous *C. chinensis,* the optimal embedding parameters were (*E*, *τ*, *T_P_*) = (4, 3,4). For *C. maculatus*, the optimal (*E*, *τ*, *T_P_*) were (4, 1, 4) and (3, 3, 1) for polygamous and monogamous treatments, respectively. Subsequent CCM identified significant interspecific interactions; cross‐mapping with the optimal embedding parameters showed significant convergence in all combinations of species and treatments under at least some time lag *l* (Figure 3). The optimal time lag in cross‐mapping *l* for *C. chinensis* xmap *C. maculatus* (i.e., the influence of *C. maculatus* on *C. chinensis*) was − 3 in the polygamous treatment and − 4 in the monogamous treatment. For *C. maculatus* xmap *C. chinensis*, *l* was 0 for polygamous treatment, and monogamous treatment showed comparable peaks at *l* = 0 and − 3, with the latter producing slightly higher forecast skill. In other words, *C. maculatus* abundance affected the abundance of *C. chinensis* with 3 or 4 weeks of time lag, whereas *C. chinensis* abundance affected *C. maculatus* immediately or 3 week later. The results of cross‐mapping were mostly similar when *E* or *τ* were slightly changed (± 1) (Figure S3). In addition, the cross‐mapping from our data to that of Kishi et al. (2009) indicated the false positive be < 0.05 (Figure S4). Because different parameter sets were estimated for polygamous and monogamous treatments for both species, direct within‐species comparison of S‐map coefficients between treatments was difficult. Therefore, for each treatment of each species, we performed two S‐map analyses with “optimal” (i.e., optimized for the focal treatment) and “suboptimal” (optimized for the other treatment in the focal species) parameters. In all S‐map analyses, optimal *θ*, the measure of nonlinearity, was 1.6–6.5 (Figure S5), confirming nonlinear dynamics. The subsequent S‐map analysis with optimal embedding parameters indicated that the population growth of polygamous *C. chinensis* was more strongly suppressed by *C. maculatus* than monogamous ones, with S‐map coefficients for polygamous *C. chinensis* significantly smaller than those for monogamous ones (|*t*_88_| = 8.23, *p* < 0.0001; Figure 4a, Table S1). However, this difference was not robust to embedding parameters (Figure 4a, Table S1). The influence from *C. chinensis* to *C. maculatus*, estimated with optimal embedding parameters, showed more varying and larger (often positive) effects in polygamous than in monogamous treatment (variance: *F*_40,60_ = 83.21, *p* < 0.0001; mean: |*t*_40.65_| = 3.83, *p* = 0.002, Figure 4b, Table S2). This difference in variance was not robust to the embedding parameters, but between‐treatment comparisons in mean were all significant or marginally significant (Figure 4b, Table S2). Time series of the estimated coefficients showed neither a clear increasing or decreasing trend (Figure S6).

## 4 DISCUSSION

We performed interspecific competition experiments involving populations that had evolved either under polygamy or monogamy for 17 generations to examine how past evolutionary histories affect the demographic outcomes of competition. Survival analysis found a marginally significant change of extinction risk ratio along time between experiments involving polygamous and monogamous lines. EDM analysis found optimal embedding parameters different between treatments. These results suggest the evolution of demographic interspecific interactions, including reproductive interference, though not highly conclusive. Below, we discuss the implications and limitations of the findings.

The optimal embedding parameters (*E*, *τ*, *T_P_*, and *l*) were different between the evolutionary treatments (polygamy vs. monogamy) in both species, implying the evolution of interspecific interactions. Particularly, because all competition experiments involved the same *C. maculatus* strain, the difference in embedding parameters for *C. maculatus* between the treatments is attributable to the evolutionary history of their competitor, *C. chinensis*. Interestingly, the optimal time lag in the influence from *C. chinensis* to *C. maculatus* was different between the treatments, with no time lag in polygamy and three weeks lag in monogamy (Figure 3). Also, the peak of the forecast skill along the time lag was monomodal for polygamous treatment but that for monogamous treatment was multimodal, with comparable peaks at 0‐ and 3‐week time lag. The time lag difference suggests differential timescales at which interspecific interactions exert their demographic effects. Behavioral mechanisms of interspecific interactions vary in the time lag from the behavioral interaction to the occurrence of its demographic effect (Kawatsu & Kishi, 2018). In seed beetles, reproductive interference shortens adult *C. maculatus* female longevity. Therefore, reproductive interference likely shows no or short time lag in weakly census data of adult beetles. The effect of larval resource competition, on the other hand, is expected to show 3‐ or 4‐week time lag, because the number of reproducing adults in the previous generation (~ 3 or 4 weeks ago) should affect the intensity of larval resource competition experienced by the focal generation (Kawatsu & Kishi, 2018). The comparable peaks at no and three‐week lags for the monogamous treatment thus implies comparable importance of resource competition and reproductive interference, whereas no time lag for the polygamous treatment suggests predominant importance of reproductive interference. This result is consistent with the behavioral assay where polygamous *C. chinensis* showed stronger reproductive interference than monogamous ones (Kyogoku & Sota, 2017).

The strongest negative influences from *C*. *chinensis* to *C. maculatus* were estimated with no time lg (*l* = 0) in both treatments. This strong negative effect could be underlain by reproductive interference, which should exert its demographic effect with little time lag (Kawatsu & Kishi, 2018; see above). However, mean of S‐map coefficients was larger in polygamous than in monogamous treatment (Figure 4b, Table S2), and thus we did not find the evidence for the prediction that polygamous *C. chinensis* persistently exert stronger negative effect than monogamous ones on *C. maculatus* via reproductive interference. Furthermore, S‐map coefficients suggested that *C. chinensis* abundance frequently had facilitative effects on *C. maculatus*. S‐map quantifies combined effects of all interspecific interaction mechanisms. A possible explanation for the positive effects from *C. chinensis* to *C. maculatus* is the facilitative effect of larval density on their survival at relatively low‐density regimes (Giga & Smith, 1981), such as by modification of the abiotic environment (Allee, Emerson, Park, Park, & Schmidt, 1949; Utida, 1998). Indeed, particularly strong positive effects were observed at early stage of competition, when the density was low (Figure S6). Thus, the varying influence from *C. chinensis* to *C. maculatus* that range from negative to positive may reflect multiple interaction mechanisms.

The demographic effect of *C. maculatus* on *C. chinensis*, estimated by S‐map with optimal embedding parameters, suggested that the population growth of *C. chinensis* was suppressed more strongly by *C. maculatus* in polygamous than in monogamous treatment (Figure 4a). This result appears consistent with the initially high relative extinction risk for polygamous *C. chinensis* (Figure S1). The estimated time lag of 3 or 4 weeks for this effect corresponds to their generation time, suggesting larval resource competition as the underlying mechanism (Kawatsu & Kishi, 2018). Larvae of *C. maculatus* are more competitive than those of *C. chinensis* (Kishi et al., 2009), and abundant oviposition by *C. maculatus* females in the previous generation may have intensified larval competition and therefore decreased *C. chinensis* abundance. Yet, we note that the result of S‐map for the influence of *C. maculatus* to *C. chinensis* was not robust to embedding parameters and not conclusive. Our EDM analysis was based on two replications from each treatment, with relatively short time series. More replications might have led to more conclusive results.

Though we used the same experimental settings with those of Kishi et al. (2009), in which *C. chinensis* exerted reproductive interference effectively and necessarily outcompeted *C. maculatus*, *C. chinensis* was conversely outcompeted by *C. maculatus* in all trials of our study (Figures 1 and 2). We have no definite explanation for this result, yet it is likely that some characters related to competitive ability of *C. chinensis* lines and/or the *C. maculatus* strain differed from those used in Kishi et al. (2009). It was possible that the *C. chinensis* evolutionary lines had accumulated deleterious alleles due to their small effective population sizes during the experimental evolution and had unusual characters. However, in a parallel competition experiment using the jC‐F strain of *C. chinensis* which was used in Kishi et al. (2009), *C. chinensis* was again excluded by *C. maculatus* in all five replicates (D. Kyogoku and T. Sota unpublished). Therefore, the characteristics of *C. maculatus* used in our study, such as larval competitive ability, may have been different from those of *C. maculatus* larvae used in Kishi et al. (2009). Indeed, the *C. maculatus* strain that we used, hQ, seems evolutionarily labile in terms of larval competition ability (Mano & Toquenaga, 2008).

In the survival analysis, the relative likelihood of extinction in a unit time of polygamous versus monogamous lines changed over time (Figure S1), whereas the peak of polygamous *C. chinensis* lines extinctions (weeks 14–24) preceded that of monogamous lines (weeks 17–31), the two remaining polygamous lines that survived this phase persisted for more than 35 weeks. In other words, the dynamic trajectory of interspecific competition involving polygamous lines may have stochastically resulted in either quick extinction or somewhat long persistence. This result may imply that the past evolutionary history influenced the population dynamics of interspecific competition in an unexpected way.

We predicted that the evolution of stronger reproductive interference under polygamy than under monogamy, which was previously found in a behavioral assay (Kyogoku & Sota, 2017), would translate into the demographic dynamics of interspecific competition at population level. This prediction was partly supported by our results. The time lag of interspecific interactions estimated by CCM implied the evolutionary changes in the relative importance of larval resource competition and adult reproductive interference, as we expected. However, the intensity of interspecific interactions estimated by S‐map was not fully in accordance with the expectation of stronger reproductive interference by polygamous than monogamous *C. chinensis*. Furthermore, the extinction of *C. chinensis* was slightly quicker in competition trials involving polygamous than monogamous *C. chinensis*, on the contrary to our prediction, though the difference was not significant. These results imply complex relationship between phenotypic evolution and demographic dynamics of competition. For example, correlated evolution of multiple traits (e.g., due to constraint or independent evolutionary responses to the environment) may have affected interspecific interactions other than reproductive interference. Alternatively, the intensity of reproductive interference may not have simple linear relationship with demographic dynamics; it might be possible that strong reproductive interference by polygamous *C. chinensis* destabilize the system and enhances the extinction of *C. chinensis* themselves.

## 5 CONCLUSIONS

We examined the competitive dynamics of *C. chinensis* and *C. maculatus* after manipulating the evolutionary history of *C. chinensis* under different mating regimes and tested the hypothesis that sexual selection has consequences for competitive dynamics through its effect on the strength of reproductive interference, a dominant mode of interspecific interaction in our system (Kawatsu & Kishi, 2018; Kishi et al., 2009). Although our results were inconclusive and did not fully support our hypothesis, we found that past evolutionary history affected some properties of competitive dynamics. This result adds to the growing literature on the side effects of sexual selection on ecological dynamics (Lumley et al., 2015; Rankin, Dieckmann, & Kokko, 2011; Takahashi, Kagawa, Svensson, & Kawata, 2014). Though we focused on reproductive interference here, sexual selection can affect ecological dynamics via other mechanisms. For example, harmful effects of sexually selected male traits can reduce conspecific female fitness (Gay et al., 2011; Rice, 1996), and female mate choice for males in good condition can increase population fitness by purging unfit alleles (Lumley et al., 2015). Further investigations will deepen our understanding of the role of sexual selection in determining ecological dynamics.

## CONFLICT OF INTEREST

Authors declare no conflict of interest.

## AUTHOR CONTRIBUTIONS

D. K. conceived the study, conducted the experiments and data analyses, and drafted the paper. T.S. contributed to the experimental design. M.K. contributed to the data analysis. All authors prepared the manuscript.

## DATA AVAILABILITY STATEMENT

The data underlying this study is available on Dryad (DOI:https://doi.org/10.5061/dryad.96q5g80).
