# Supplementary material for: Does past evolutionary history under different mating regimes influence the demographic dynamics of interspecific competition?
Source: Ecol Evol. 2019 Jul 5;9(15):8616–24. doi: 10.1002/ece3.5397 (PMC6686342; doi:10.1002/ece3.5397)
Supplement: Supplementary file 1 [file ECE3-9-8616-s001.docx]

**Does past evolutionary history under different mating regimes influence the demographic dynamics of interspecific competition?**

Daisuke Kyogoku*, Michio Kondoh, Teiji Sota

*d.kyogoku@gmail.com

**Supplementary methods**

Details of EDM analysis

EDM is based on Takens’ theorem, which claims that a time series of a single variable (e.g., population dynamics of one species) contains sufficient information on the mathematical properties of the whole dynamic system (e.g., population dynamics of two competing species). The state space reconstruction by a single time series uses lagged coordinate embedding: ***x_t_*** = {*x*(*t*), *x*(*t* − *τ*), *x*(*t* − 2*τ*),…, *x*(*t* − (*E*−1)*τ*)}, as explained in the main text. Because a single replicate of our competition experiment consisted of only about 20 census points, we used both replicates from the same treatment to reconstruct the state space (i.e., dewdrop regression; Hsieh et al. 2008; Clark et al. 2015). For the analysis, we scaled the population dynamics by subtracting the mean and then dividing by the standard deviation.

We first determined the embedding dimension *E* by simplex projection. In this simplex projection, we varied the number of time steps to predict, *T_P_*, as well as *E*. For example, when *T_P_* = 3, *x*(*t* + 3) is predicted by ***x_t_***. We defined optimal *E* and *T_P_* as those yielding the maximum forecasting skill, evaluated by the correlation coefficient between the predictions and observations.

Using the optimal *E* and *T_P_*, we examined the demographic interactions between *C. chinensis* and *C. maculatus* by performing cross mapping. For example, when the abundance of *C. chinensis* affects the demographic dynamics of *C. maculatus*, the census data of *C. maculatus* contains information on the past abundance of *C. chinensis*. In other words, if one variable influences another, the past state of the influencing variable can be estimated by the time series of the influenced variable (note that the directions of prediction and causation are reversed). CCM analysis exploits this principle to examine the causal relationships among variables (Sugihara et al. 2012). We performed CCM analysis by varying *l*, the time lag for prediction, in [−8, 0], and we evaluated CCM by nonparametric bootstrap. Specifically, we randomly chose *E* + 1 data points from the reconstructed state space, performed cross mapping (i.e., predicted the abundance of the other species) and evaluated the forecast skill by the correlation coefficient. We repeated this 1,000 times and produced the distribution of the forecast skill with the minimum data (or library) size. The convergence of the forecast skill was considered significant when the forecast skill with the maximum library size (i.e., all data) was not included in the 95% confidence interval under the minimum library size. When interspecific interaction was significant, we chose the best *l* on the basis of the correlation coefficient under the maximum library size.

Finally, we quantified the intensities of interspecific interactions by applying S-map to multivariate reconstructed state spaces. S-map analysis predicts the future state of the system by regression in state space, where data points are weighted depending on their distance from the predictee. For multivariate S-map analysis, we replaced the oldest component of ***x_t_*** (i.e., *x*(*t* − (*E* − 1)*τ*)) with the census data of the other species, with the optimal *l*: {*x*(*t*), *x*(*t* − *τ*), *x*(*t* − 2*τ*),…, *x*(*t* − (*E*−2)*τ*), *y*(*t* + *l* + *T_P_*)}, where *y* is the value of the variable influencing *x*. The optimal distance-weighting parameter *θ*, which gives the maximum forecast skill, was determined by varying the parameter in the range of [0.5, 10] with 0.1 intervals. S-map reduces to ordinary linear regression when *θ* = 0, and large *θ* indicates strong nonlinearity (i.e., state-dependence of the system).

**References**

Clark AT, Ye H, Isbell F, Deyle ER Cowles J, Tilman GD, Sugihara G (2015) Spatial convergent cross mapping to detect causal relationships from short time series. Ecology 96:1174–1181. doi:10.1890/14-1479.1.sm

Hsieh C, Anderson C, Sugihara G (2008) Extending nonlinear analysis to short ecological time series. Am Nat 171:71–80. doi:10.1086/524202

Sugihara G, May R, Ye H, Hsieh C, Deyle E, Fogarty M, Munch S (2012) Detecting causality in complex ecosystems. Science 338:496–500

**Supplementary results**

**Table S1**. Multiple comparisons for S-map coefficients quantifying the influence from *C. maculatus* to *C. chinensis*. *P* values were adjusted by the Holm-Bonferroni method, and the degrees of freedom for *t* test were adjusted by Welch’s method for comparisons with significant variance difference.

| Comparison pair | Variance | Mean |
| --- | --- | --- |
| Optimal polygamy vs. suboptimal polygamy | *F*_40,32_ = 0.120  (*P* < 0.0001) | \|*t*_38.19_\| = 0.221  (*P* = 1) |
| Optimal polygamy vs. optimal monogamy | *F*_40,48_ = 0.864  (*P* = 1) | \|*t*_88_\| = 8.22  (*P* < 0.0001) |
| Optimal polygamy vs. suboptimal monogamy | *F*_40,56_ = 0.755  (*P* = 1) | \|*t*_96_\| = 0.881  (*P* = 1) |
| Suboptimal polygamy vs. optimal monogamy | *F*_32,48_ = 7.20  (*P* < 0.0001) | \|*t*_38.04_\| = 3.23  (*P* = 0.010) |
| Suboptimal polygamy vs. suboptimal monogamy | *F*_32,56_ = 6.29  (*P* < 0.0001) | \|*t*_37.97_\| = 0.153  (*P* = 1) |
| Optimal monogamy vs. suboptimal monogamy | *F*_48,56_ = 0.874  (*P* = 1) | \|*t*_104_\| = 7.42  (*P* < 0.0001) |

**Table S2**. Multiple comparisons for S-map coefficients quantifying the influence from *C. chinensis* to *C. maculatus*. *P* values were adjusted by the Holm-Bonferroni method, and the degrees of freedom for *t* test were adjusted by Welch’s method for comparisons with significant variance difference.

| Comparison pair | Variance | Mean |
| --- | --- | --- |
| Optimal polygamy vs. suboptimal polygamy | *F*_40,44_ = 58.7  (*P* < 0.0001) | \|*t*_41.24_\| = 3.16  (*P* = 0.009) |
| Optimal polygamy vs. optimal monogamy | *F*_40,60_ = 83.2  (*P* < 0.0001) | \|*t*_40.65_\| = 3.83  (*P* = 0.002) |
| Optimal polygamy vs. suboptimal monogamy | *F*_40,56_ = 1.24  (*P* = 0.45) | \|*t*_96_\| = 3.96  (*P* = 0.0007) |
| Suboptimal polygamy vs. optimal monogamy | *F*_44,60_ = 1.42  (*P* = 0.42) | \|*t*_84.86_\| = 4.30  (*P* = 0.0003) |
| Suboptimal polygamy vs. suboptimal monogamy | *F*_44,56_ = 0.021  (*P* < 0.0001) | \|*t*_58.99_\| = 2.21  (*P* = 0.061) |
| Optimal monogamy vs. suboptimal monogamy | *F*_60,56_ = 0.015  (*P* < 0.0001) | \|*t*_57.57_\| = 1.37  (*P* = 0.18) |

**Fig. S1.**

Schoenfeld residual plot along time to examine the proportionality assumption of the Cox model. The solid line is the regression line, and dashed lines represent confidence intervals (2 SE). If hazard functions are proportional between polygamous and monogamous lines regardless of time, the slope of the regression should be zero. *β* is the parameter of the Cox model.


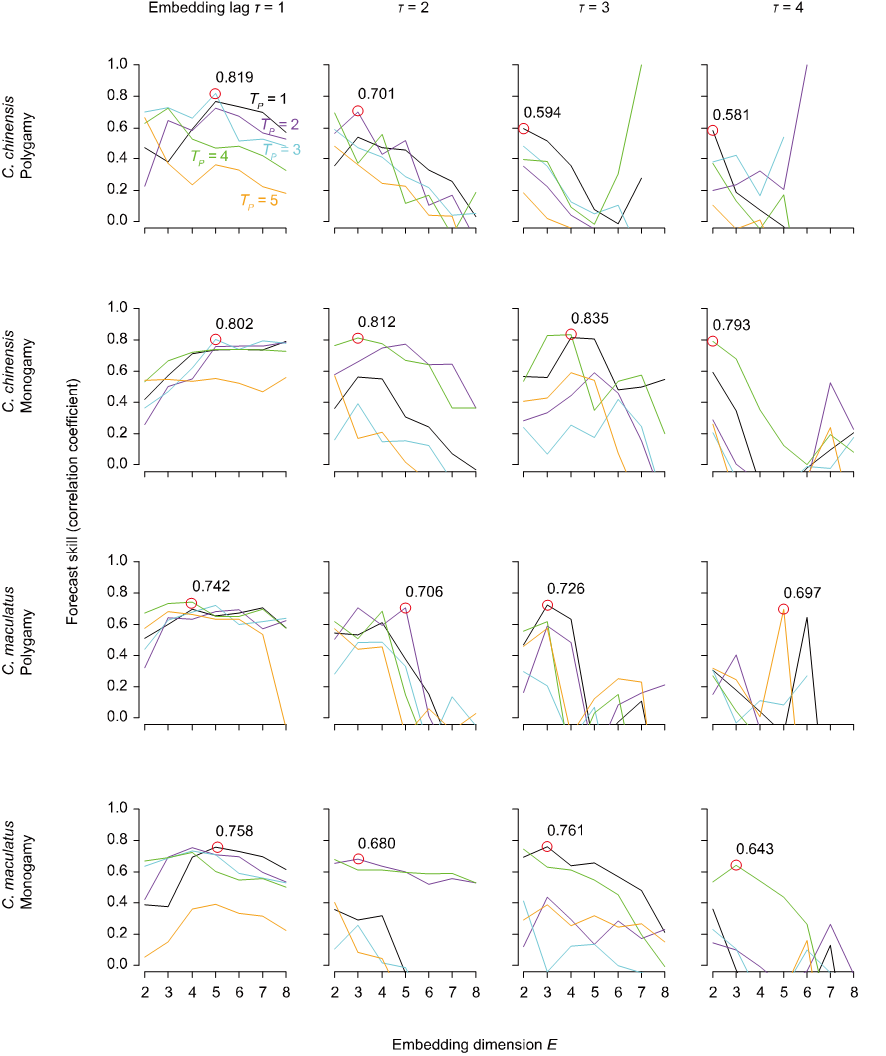


**Fig. S2.**

Univariate simplex projection. In each panel, black, purple, blue, green, and orange lines give the results regarding the time steps to predict *T_P_* = 1, 2, 3, 4, and 5, respectively. Red circles indicate the combination of *T_P_* and the embedding dimension *E* that shows the highest forecast skill in a panel, and the associated numbers indicate the forecast skill. Sudden and sharp increases in forecast skill with high *E* were considered as anomaly (e.g. *C. chinensis* of polygamous treatment, *T_P_* = 4, *E* = 7).


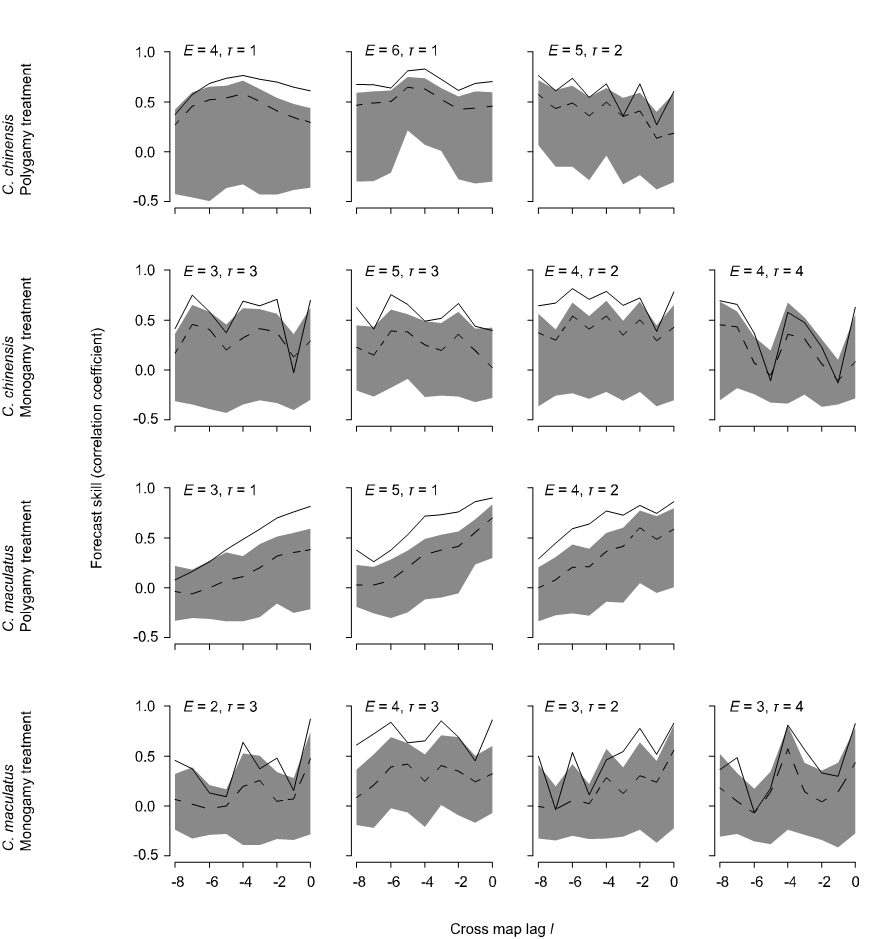


**Fig. S3.**

Convergent cross mapping with embedding dimension *E* and embedding lag *τ* that deviate from their optimal values.


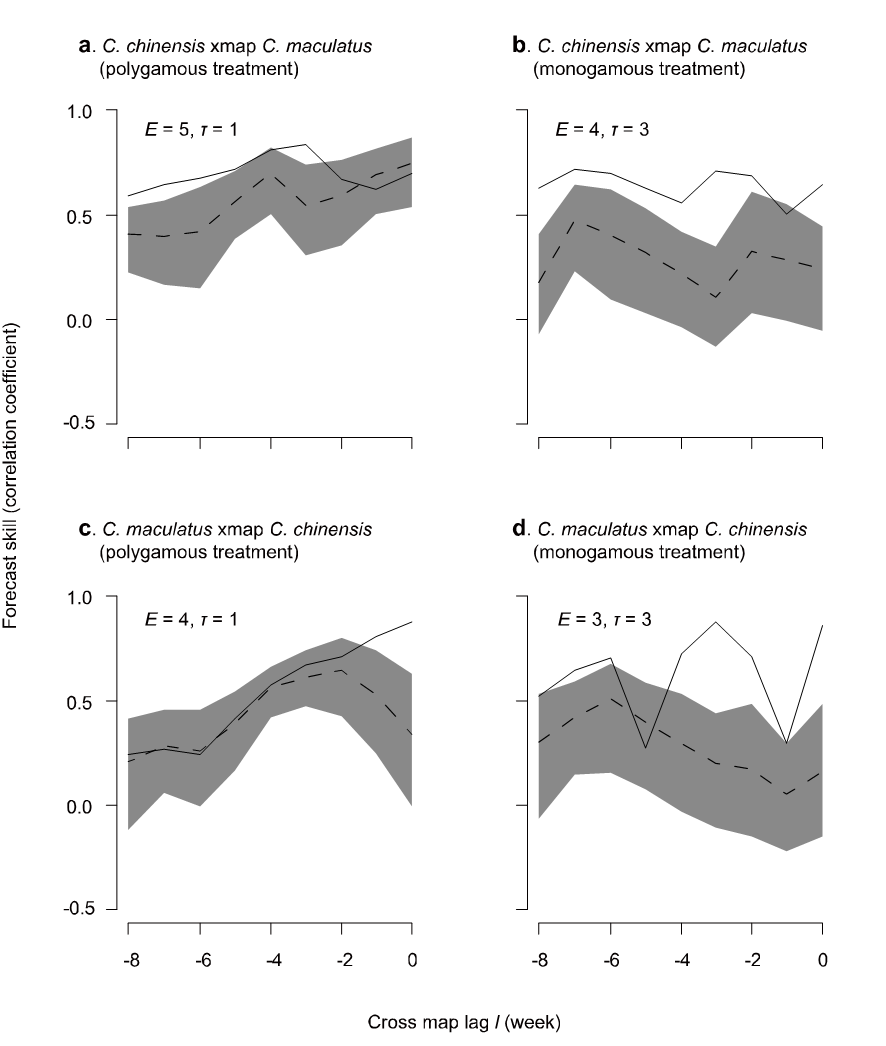


**Fig. S4.**

Convergent cross mapping with natural surrogate data (from Kishi et al. 2009). Solid lines give the forecast skill when our data is mapped to ours under a given cross map lag *l* (i.e. identical to the solid lines in Fig. 3). Dashed lines give the median forecast skill when our data is mapped to that of Kishi et al. Gray areas give 95% confidence interval of the forecast skill of cross mapping our data to that of Kishi et al.


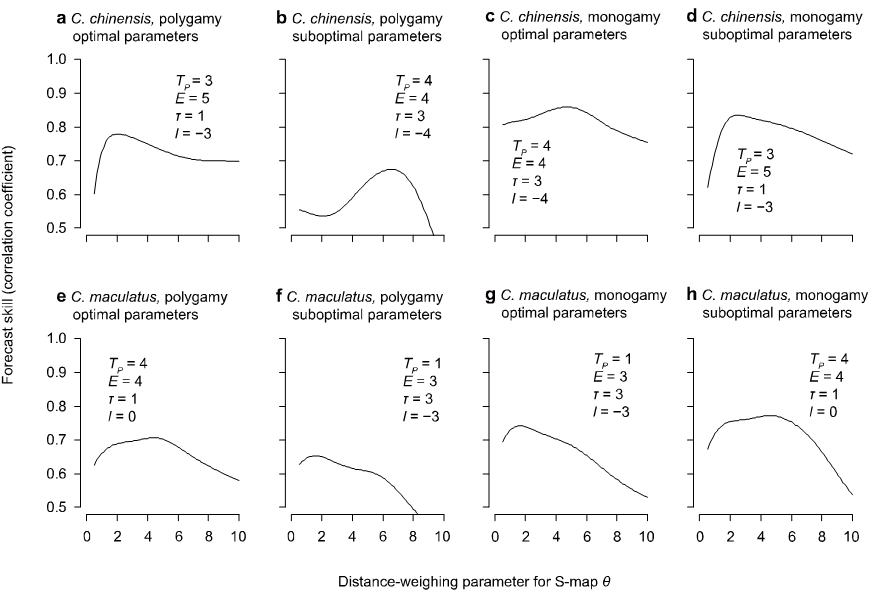


**Fig. S5.**

Distance-weighing parameter (*θ*) dependence of the forecast skill of S-map. Analysis for each species from each treatment was performed with two embedding parameter sets (*T_P_*, *E*, *τ*, *l*); one optimized for the focal species of the focal treatment, the other (suboptimal) one was that optimized for the focal species of the other treatment.


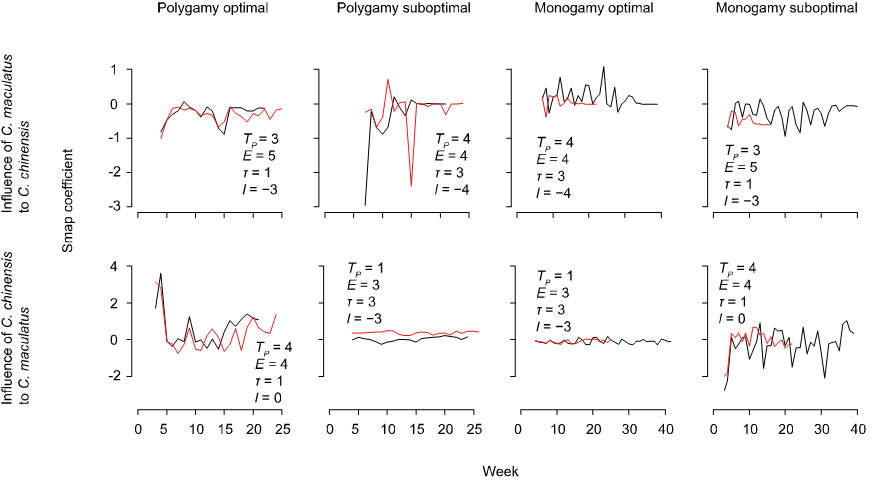


**Fig. S6.**

Time series of S-map coefficients for interspecific interaction. Red and black lines represent independent replicates. Analysis for each species from each treatment was performed with two embedding parameter sets (*T_P_*, *E*, *τ*, *l*); one optimized for the focal species of the focal treatment, the other (suboptimal) one was that optimized for the focal species of the other treatment.
